# Are all electrons the same? Evaluating support for local transmission lines through an experiment

**DOI:** 10.1371/journal.pone.0219066

**Published:** 2019-07-17

**Authors:** Sanya Carley, Stephen Ansolabehere, David M. Konisky

**Affiliations:** 1 Paul O’Neill School of Public and Environmental Affairs, Indiana University, Bloomington, Indiana, United States of America; 2 Department of Government, Harvard University, Cambridge, Massachusetts, United States of America; Institute for Advanced Sustainability Studies, GERMANY

## Abstract

A crucial factor in U.S. energy infrastructure development is the degree to which citizens that reside near a development support or oppose the project. While the literature offers several explanations about what shapes individuals’ perceptions of energy projects, few have considered the importance of cognitive heuristics and the law of contagion. Here, we employ a survey experiment on a nationally-representative sample of 2,000 respondents to test whether knowing which energy resources connect to a high-voltage transmission line dictate support for the line. Results reveal that information about the source of electricity is fundamentally important. If a transmission line is said to carry electricity from a solar and wind development, a respondent is significantly more likely to support development of the line. If the line instead is said to carry electricity from a natural gas or coal plant, respectively, respondents are significantly less likely to support it. This study has implications for energy infrastructure development, messaging, and public acceptance of energy projects.

## Introduction

The American Society of Civil Engineers recently assigned the U.S. transmission system, and energy infrastructure more generally, a D+ grade in their 2017 infrastructure scorecard [1]. This grade clearly reflects a growing need for new and updated U.S. transmission infrastructure.

Most of the approximately 640,000 miles of high-voltage transmission lines in the United States were built in the 1950s or 1960s and were designed to last about 50 years [1]. These outdated lines not only degrade with use and time, but they often carry electric loads that are far heavier than their designed capacity. New upgrades are also needed to maintain appropriate levels of grid resiliency, especially against catastrophic events as the rate of weather-related and other high-impact disasters rises [2, 3]. In addition, as the United States continues to invest in new renewable energy and natural gas power plants—with 869 new renewable generators and 364 natural gas generators planned for construction between 2018 and 2022 [4]—more transmission infrastructure will be needed to integrate these resources into the grid [5], with an estimated price tag of at least $5 trillion [6].

Financing new infrastructure developments is clearly a critical aspect of modern electricity operations, but perhaps even more so is the siting process and the degree to which communities that will host the projects support or oppose their development. Without support from local communities, it is immensely difficult if not impossible to develop new infrastructure. Transmission lines are often met with opposition, sometimes fierce opposition, which results in project delays and increases in costs [7, 8, 9]. As the United States—as well as other countries across the world—increasingly builds new renewable energy and natural gas facilities, the need for new transmission continues to grow [9, 10, 11]. Public opposition is considered by some to be a critical inhibitor to successful siting of new infrastructure to satisfy these growing transmission needs [11].

For this reason, it is important to understand the dynamics behind public support or opposition to new high-voltage transmission lines. The extant literature has provided helpful insights regarding the role of individual-level factors in shaping support for a project, such as one’s place-based attachments, beliefs about the environment, familiarity with energy types, or trust in the agents making decisions [7, 12, 13, 14, 15, 16, 17, 18, 19, 20, 21, 22]. Researchers have also studied the role of perceptions about project-related factors such as specific details about the project, and the degree to which such details trigger concerns about risks or perceptions of benefits [23, 24]. The present study extends the literature on project-specific factors. Building on insights from social psychology about how individuals use cognitive heuristics to form perceptions, we study whether an individual’s support for a transmission line is affected by information about the source of energy used to generate the electricity that it carries.

More specifically, we employ an embedded survey experiment to a nationally-representative sample (n = 2,000) of respondents to test whether knowing the source of electricity that is carried in the lines—specifically, renewables, natural gas, or coal—affects individuals’ level of support for line construction near their home. Of course, an electron generated by one source such as a coal-fired power plant is no different than an electron generated by another source such as a solar photovoltaic panel. The actual transmission line should look and function the same, regardless of the sources of electrons that it carries. However, we posit that if someone knows that an electron was generated via one energy source or another, it will affect how they perceive the transmission line. Underlying this relationship, we think, is an intuitive contagion heuristic [25] by which people attach their perceptions of the energy source (e.g., wind is relatively “clean” and coal is relatively “dirty”) to the transmission line.

Consistent with our expectations, we find that, when one learns that a transmission line carries electricity produced by a renewable energy technology such as solar or wind, they are significantly more supportive of it. Alternatively, if one learns that it carries electricity produced at coal or natural gas plants, they are significantly less likely to support it. These results hold to variety of statistical tests and robustness checks, and remain present when controlling for a large suite of individual-level variables and other factors that the literature has previously shown to predict attitudes toward energy projects generally and transmission lines specifically. We discuss the implications of these results in the conclusion.

### Public support and opposition of energy infrastructure

Studies of public attitudes toward energy have identified a number of important determinants of how people think about infrastructure projects. Traditionally, researchers have considered a broad set of individual-level factors as being central. Several studies, for example, find that support for transmission lines is often hindered by an individual’s sense of place and the manner in which this place-attachment facilitates place-specific identities [7, 12, 13]. These place-based attachments often explain the “Not in My Backyard” sentiments that arise around other types of infrastructure as well, such as wind farms [26]. Scholars have also demonstrated the importance of personal beliefs, such as one’s worldviews [27, 14] and political orientation [15], which more generally have been found to explain attitudes toward climate change [28, 29, 30, 31] and environmental policy [27, 32]. Although few have tested the relationship between environmental beliefs and support for energy infrastructure, and specifically transmission lines, there is a large body of literature within the ecological economics and sustainable behavior fields that find that environmental beliefs are a key factor in determining adoption, and willingness to pay or accept energy related goods and technologies, such as electric vehicles [16], recycled paper and other household products [17], and green electricity certificates [18].

Research has focused on other individual-specific factors as well, such as the role of familiarity with specific energy technologies. Some, for example, have found that a key driver of energy infrastructure attitudes is one’s prior familiarity with the type of technology in question, such that higher levels of familiarity with high voltage transmission lines leads to greater support of the technology [19]. One’s trust toward government or industry may also dictate support for an energy infrastructure project [7, 20], since those that do not trust the government or industry to make good decisions or sound policy will be less inclined to believe that the government or industry will pursue infrastructure developments adequately. Trust in these entities can also drive perceptions of risk associated with a specific project [21]. Trust also affects one’s sense of political efficacy and ability to engage in the siting and development process, which in turn also affects support [12, 23, 19].

A second, more recent stream of work, emphasizes project-specific factors such as the details of the project itself (e.g., location, integration into the landscape, and compensation to landowners) [33, 9], beliefs about the fairness of the siting process [34], and perceptions of risk [13] and benefits [9, 35] related to the project. Project details could be important because they reveal possible environmental, economic, aesthetic, or social impacts [7, 36, 12, 19]. This line of reasoning is consistent with recent work that emphasizes that people’s opinion toward energy is based on their perceptions of the attributes of energy sources [37, 38, 22]. Specifically, this work shows that perceptions of the local environmental harms and economic costs that people associate with different modes of electricity generation are central to their views. For example, people do not favor wind because it is wind power or disfavor coal because it is coal. Rather, their preferences toward these sources are based on their views that wind is cleaner and less expensive than coal. Research has further demonstrated that this structure of public opinion toward energy—that is, that people’s attitudes are shaped by their perceptions of the energy resources’ attributes—extends to energy siting [15] and policy preferences [15, 39, 40, 41].

This emerging body of research suggests that the attributes of energy are an important project-specific factor that may affect how people form opinions about infrastructure projects. In other words, individuals make evaluations about what type of energy they prefer, including perhaps their preferences toward specific manifestations of that energy as embodied in infrastructure projects, through an assessment of its attributes. This is not to suggest, however, that individuals necessarily engage in a formal process of evaluation. Rather, it is possible that they rely on mental shortcuts or heuristics when making judgments.

Ideas developed within social psychology offer some insights as to the cognitive link people make between their perceptions of energy types and the degree to which they support, or not, a specific infrastructure project. Foundational work in social psychology on the perceptions of risk has shown that people rely on a number of different heuristics when evaluating technologies [42]. Project-related factors, as communicated or understood by an individual, could trigger cognitive, affective responses about the possible benefits or risks associated with an energy project without a full and conscious weighing of net costs [43, 44]. These affected responses are connected to the feelings that one attributes to different energy technologies. These sentiments could lead one to assign more or less risk to, or sense of opportunity or concern about, a project based on intuitive, automatic reasoning [44].

Indeed, several studies have found that affective responses emerge when one is asked about different energy technologies, including positive feelings when asked about solar and negative feelings when asked about nuclear [45, 46]. One study found that individuals are willing to accept less compensation for wind turbines located in their home county if they believe that wind energy is “green,” relative to those that do not carry this perception [47]. One study focused on high-voltage transmission lines and tested whether affective responses led individuals to be more supportive of a transmission line if they knew that grid expansion was necessary for renewable energy development as part of the broader energy transition [9]. This study not only confirms that affective responses exist—i.e., those that see greater benefit to renewable energy are more accepting of transmission lines—but also demonstrates that the affect is much stronger when the project is located locally than if it is instead framed in general terms. The latter finding reaffirms insights made by others that, if one is aware that a transmission line will be located in close proximity to his or her home, the importance of project-specific factors, such as possible risks and benefits, likely grows [19, 8].

Of particular relevance for this study is the magical law of contagion, or what is often referred to as the intuitive contagion heuristic. The central idea is that when two entities come into contact, they exchange properties, or what social psychologists have called “essences” [25]. Empirical research has demonstrated that contagion beliefs are widespread, especially in instances when one of the objects is perceived to be disgusting (see, e.g., [48, 49]). That is, when an individual has an aversion to a specific thing, the sentiments of disgust not only affect one’s behavior toward it, but it can also affect one’s reactions to other things that come into contact with it [50, 25, 51, 52, 53]. This so-called contagion can lead an individual to intuitively reject the related thing as a cognitive heuristic, rather than thoroughly weighing all benefits and risks and seeking out detailed information about it. The inverse should also be the case. That is, when an individual ascribes positive views toward an object, they may carry over into another object with which it comes into contact. This theory has been used to explain why individuals are generally not supportive of recycled wastewater [25], genetically modified foods [54], and “if it’s yellow, let it mellow” flushing practices [55], among others.

In the case of transmission lines, the focus of this study, we posit that people’s level of support may be connected by contagion to their perceptions of the source of energy—and the embodied attributes thereof—used to generate the electricity that the lines carry. Existing research shows that individuals’ perceptions of energy sources and related infrastructure is strongly affected by their views of how clean or dirty it is as a source of electricity generation [15, 37]. Therefore, we expect that individuals will be more supportive of transmission lines if they believe the lines carry renewable energy-derived electricity rather than fossil fuel-derived electricity. To date, there is not significant empirical inquiry into this subject beyond those studies reviewed above; no studies, to the authors’ knowledge, test directly the possibility of contagion and cognitive heuristics in the context of transmission line siting, as we do here.

## Methodological approach

In this analysis, we employ data collected from an original public opinion survey with an embedded experiment. The survey was administered in October 2017 by YouGov, a private polling and marketing firm, with whom we complied by their terms of service. The survey was administered on a random and representative sample of 2,000 individuals located across the U.S. To generate this sample, YouGov creates a population-based target sample, and then draws a random set of respondents from their opt-in pool of participants to match the target population. For their participation in the survey, respondents were offered points in compensation, which they can accumulate to cash in for gift cards.

The survey vignette was placed toward the end of the survey, following questions about general support for different energy and infrastructure types, and other related questions, as we discuss below. When the respondents reached the point of the survey in which they were presented with the vignettes, we randomly assigned each respondent into either a control group or one of three treatment groups. No survey respondent saw more than one vignette. The respondents were not aware, however, that they were given a random assignment, nor that the content that we provided to them was any different than content seen by another participant. The control group was told “To meet growing electricity demand in your community, it will be necessary to build new transmission lines in your area that connect to new sources of electricity generation”. The first treatment group was given this same information as well as the following sentence, which was tacked on to the end of the above statement: “These new sources consist of several solar and wind farms that have been built to generate electricity.” The second and third treatment groups were instead told that the new sources were natural gas and coal-fired power plants, respectively. Respondents were then asked, “Would you support or oppose a decision to build these transmission lines to connect these new sources of electricity?,” and provided a Likert scale set of responses to gauge level of support, from 1 (strongly oppose) to 5 (strongly support). This last question serves as our dependent variable and the assignment to treatment groups serves as the primary independent variable.

The phrasing of the experiment was especially important, and required care in three specific ways. First, the description of the transmission line, and the energy facility from which the electricity was sourced, had to be entirely neutral and unbiased. Second, we used the phrase “in your area” when discussing the new transmission line, so as to suggest that the line will be in proximity to one’s home. Previous studies have found that proximity, either real or perceived, to an energy facility can affect one’s support [8, 56]. Third, we phrased the question as one of support or opposition, rather than “acceptance,” since the literature argues that the former is more precise and accurate in this type of study [57].

This research was approved by the Office of Research Compliance at Indiana University, under protocol number 1607609309. In accordance with this protocol, informed consent was provided by all study participants.

## Results

To analyze the data, we confirm that the four groups are balanced on all observable and relevant demographics, which we find to be the case and present in Table 1. Our primary interest is in whether the mean value for each variable in each treatment group, respectively, differs from the mean value for that variable in the control group. We test this directly with two-tailed t-tests. We present the p-values under each treatment group. As the results reveal, the groups are balanced on all demographic variables save one: gender between the renewable energy treatment group and the control group. The difference in means between the treatment and the control groups on the female variable is six percentage points, a difference that is statistically significant at the ten percent significance threshold. We also test the difference in means across all four groups, as estimated through a one-way Analysis of Variance (ANOVA) and presented in the last column of the table. Overall, these results confirm that the randomization of respondents into groups was successful, with the minor exception of gender balance in the renewable energy treatment group, and that the respondents do not significantly differ along observable demographics across groups.

**Table 1:** Demographic statistics by control and treatment groups.

	Control Group			RE Treatment				NG Treatment				Coal Treatment				One-Way ANOVA
	Obs	Mean	Std. Dev.	Obs	Mean	Std. Dev.	p<	Obs	Mean	Std. Dev.	p<	Obs	Mean	Std. Dev.	p<	
Age	498	50.16	16.47	500	51.34	6.10	0.25	500	49.12	17.60	0.34	502	50.96	17.39	0.46	*F*(3, 1996) = 1.69, *p =* 0.167
Female	498	0.53	0.50	500	0.59	0.49	0.09	500	0.55	0.50	0.62	502	0.56	0.50	0.45	*F*(3, 1996) = 1.03, *p =* 0.377
Non-White Race	498	0.32	0.47	500	0.34	0.47	0.37	500	0.34	0.47	0.44	502	0.34	0.48	0.36	*F*(3, 1999) = 0.38, *p =* 0.770
Married	498	0.58	0.50	500	0.57	0.49	0.94	499	0.54	0.50	0.29	501	0.56	0.50	0.67	*F*(3, 1994) = 0.47, *p =* 0.706
Children <18	495	0.24	0.43	498	0.24	0.43	0.81	497	0.27	0.45	0.23	497	0.26	0.44	0.40	*F*(3, 1983) = 0.60, *p =* 0.612
Family Income	437	6.03	3.29	425	6.17	3.52	0.55	433	6.07	3.38	0.85	442	5.97	3.26	0.79	*F*(3, 1733) = 0.26, *p =* 0.853
Democrat	498	0.47	0.50	500	0.47	0.50	0.95	500	0.43	0.50	0.31	502	0.51	0.50	0.20	*F*(3, 1996) = 1.75, *p =* 0.156
Republican	498	0.34	0.47	500	0.33	0.47	0.75	500	0.37	0.48	0.28	502	0.30	0.46	0.17	*F*(3, 1996) = 2.04, *p =* 0.*107*F
Independent	498	0.19	0.39	500	0.20	0.40	0.77	500	0.19	0.39	0.98	502	0.19	0.40	0.99	*F*(3, 1996) = 0.04, *p =* 0.*988*F

As is common practice with experiments, our main results are a test of difference in means between the control and each respective treatment group. Since all respondents were randomly assigned to a group and the groups appear balanced on observable characteristics, and the only difference between each person’s survey experiment was a single line about the source of energy to which the transmission line connects, it is sufficient and appropriate to simply compare means.

**Fig 1:**
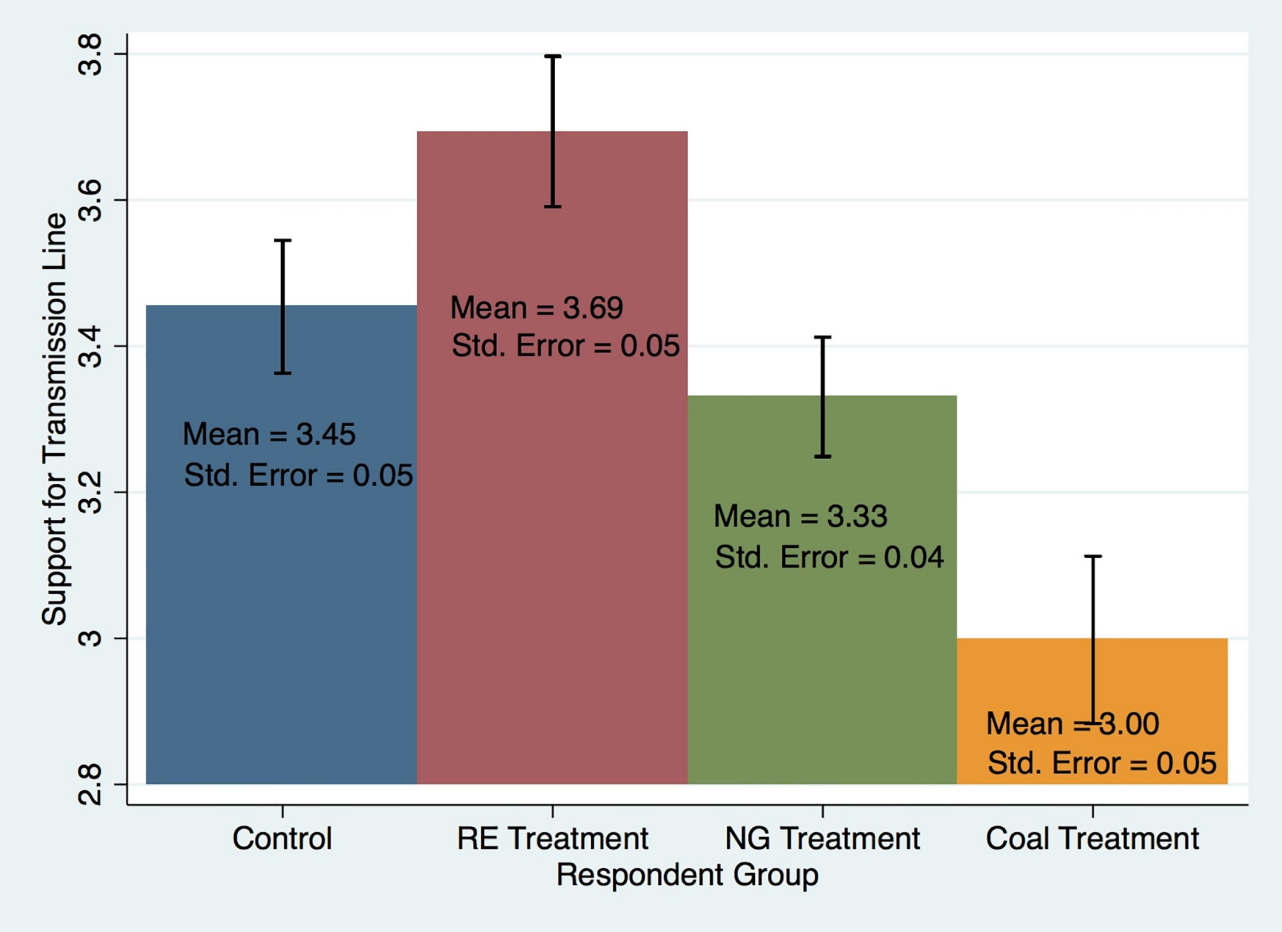
Average response to survey experiment question about support or opposition to local transmission line, by respondent group. Notes: “RE” = Renewable Energy; “NG” = Natural Gas.

The primary results are presented visually in Fig 1. The average response to the question about support for the control group was 3.45, which falls between neutral and slightly favorable on the Likert scale used to measure this variable. The average response to the question by the renewable energy, natural gas, and coal treatment groups, respectively, were 3.69, 3.33. and 3.00. These differences are large in magnitude. If one knows that an electricity line will connect to a coal-fired power plant, their support drops by 0.45, out of a 5-point scale. Alternatively, if they know that the line connects renewables, they increase their support by 0.24. The difference between the level of support for a line connecting renewables and a line connecting coal is 0.69, which corresponds to a difference of about 14 percent, based on the 5-point scale. It is important to recall that these inferences are based on deviations from the control group, so they already account for baseline support levels for transmission lines in general.

Based on a two-sample t-test, those that were told that the lines would connect solar and wind farms were significantly more likely to report support for the project than those in the control group (P<0.0007). In contrast, those in the natural gas and coal treatment groups, respectively, were significantly less likely to give support for the hypothetical transmission line, relative to the control group (P<0.0485 and P<0.000). A one-way ANOVA also confirms that the mean values for the four groups are statistically different from each other (F(3, 1988) = 33.27, r = 0.000). Information about energy types clearly affects one’s reported support for transmission lines near his/her home.

## Robustness and additional analysis

We also conduct a robustness check in the event of concerns that the random assignment process did not account for all relevant differences across the groups beyond the control or treatment conditions, even though all conditions appear to be met regarding the validity of the experiment. Specifically, we estimate an ordered probit on the dependent variable, support for the proposed transmission line, for each treatment group relative to the control group. The dependent variable is the level of support that the respondent would give to the transmission line, on a Likert scale from one to five. In these models, besides demographics, we control for other factors that the literature tends to feature as primary predictors of support or opposition, as discussed previously, such as risk aversion, trust in government and utilities, attachment to place, and environmental attitudes or worldviews. If the treatment variables maintain statistical significance, it will suggest that our experiment represents a distinctly different concept than previous theories, and adds explanatory power beyond that which the literature has already revealed.

All non-demographic variables included in the ordered probit regressions are fully defined in Table 2, with information about the corresponding alternative constructs from the literature to which each pertains. We include variables that measure one’s level of concern about climate change, level of risk aversion, trust in government and industry, place-based perceptions, environmental beliefs, general support for transmission lines, and general support for different energy technologies. We also include a standard set of demographic controls, since previous studies have found that although demographics are not the leading explanation for support or opposition, they are still important determinants [19, 7].

**Table 2:** Variable connection to construction and operationalization.

Variable	Construct	Operationalization
Support for Proposed Transmission Line	Dependent Variable	Response to question “Would you support or oppose a decision to build these transmission lines to connect these new sources of electricity?”, from 1 = strongly oppose to 5 = strongly support
Perceptions about Severity of Climate Change	Environmental and climate change beliefs	Response to question "There is a lot of talk about global warming caused by carbon dioxide emissions from human activities. Which of the following do you think best describes your view?" 1 = this is not a serious problem, 2 = more research is needed before action is taken, 3 = we should take some action now, 4 = Immediate and drastic action is necessary
Risk Aversion	Risk	Reported willingness to take risks, from 1 = very unwilling to 10 = very willing
Trust in Powerline Companies	Trust	Reported trust in companies that develop and operate electric power lines, from 1 = very low trust to 5 = very high trust
Trust in Government Factor	Trust	Factor from principal component analysis of trust in local, state, and federal government, ranging from -1.93 to 2.84
Government Makes Quality Energy Decisions	Trust	Response to question "How often do you think that government generally makes good decisions about which major energy infrastructure projects to approve?" 1 = Never, 2 = Rarely, 3 = Some of the time, 4 = Most of the time, 5 = Always
New Environmental Paradigm Factors	Environmental beliefs	Factor from principal component analysis of New Environmental Paradigm variables, ranging from -2.99 to 1.76
Perceptions of Local Environmental Quality	Place-based Perceptions	Respondent's rating of the quality of the environment in his/her local area, from 1 = poor to 4 = excellent
Perceptions of Local Economic Quality	Place-based Perceptions	Respondent's rating of the quality of the economy in his/her local area, from 1 = poor to 4 = excellent
General Support for Solar	Prior familiarity and views about a technology	Respondent reported support for building solar power plant, from 1 = strongly oppose to 5 = strongly support
General Support for Wind	Prior familiarity and views about a technology	Respondent reported support for building wind farm from 1 = strongly oppose to 5 = strongly support
General Support for Natural Gas	Prior familiarity and views about a technology	Respondent reported support for building natural gas power plant from 1 = strongly oppose to 5 = strongly support
General Support for Coal	Prior familiarity and views about a technology	Respondent reported support for building coal power plant from 1 = strongly oppose to 5 = strongly support
General Support for Transmission Lines	Prior familiarity and views about a technology	Respondent reported support for building transmission lines from 1 = strongly oppose to 5 = strongly support
Age	Demographics	Respondent’s age
Female	Demographics	Respondent identifies as a female
Non-white Race	Demographics	Respondent identifies race as non-white
Married	Demographics	Respondent is married or in domestic/civil partnership
Children under 18	Demographics	Respondent has children under the age of 18
Family Income	Demographics	Respondent’s self-reported income, ordinal variable on a 16-point scale
Republican	Demographics	Respondent identifies as a Republican
Independent	Demographics	Respondent identifies as an Independent
Treatment	Primary Independent Variable in Robustness Check (Table 6)	Indicates whether the respondent was in the treatment group, where 1 = in treatment group and 0 = in control group

Two variables included in the regression models are generated using principal component factor analysis: trust in government and the new environmental paradigm. The former includes three variables that measure trust in local, state, and the national government, respectively, as are presented in Table 3. The latter includes six New Environmental Paradigm questions that we included in our survey instrument, as designed after others’ New Environmental Paradigm survey questions [58, 59, 60] and presented in Table 4. Both sets of variables load onto a single factor. We display these principal component factor analysis results in Tables 3 and 4 along with related descriptive statistics. Descriptive statistics for the entire sample and all variables are presented in Table 5.

**Table 3:** Principal component factor analysis results for trust in government survey questions.

Variable	Factor 1
Trust in Local Government	0.847
Trust in State Government	0.911
Trust in Federal Government	0.773
Cronbach's Alpha	0.796
Eigenvalue	2.144
Kaiser-Meyer-Olkin overall measure of sampling adequacy	0.637

**Table 4:** Principal component factor analysis results for new environmental paradigm survey questions.

Variable	Factor 1
The balance of nature is very delicate and easily upset by human activities	0.713
The earth is like a spaceship with only limited room and resources	0.699
Plants and animals do not exist primarily for human use	0.642
Modifying the environment for human use seldom causes serious problems	-0.621
There are no limits to growth for nations like the United States	-0.563
Mankind was created to rule over the rest of nature	-0.715
Cronbach's Alpha	0.738
Eigenvalue	2.623
Kaiser-Meyer-Olkin overall measure of sampling adequacy	0.770

**Table 5:** Descriptive statistics.

	Mean	Std. Dev.	Min	Max
Support for Proposed Transmission Line	3.37	1.15	1	5
Age	50.40	16.90	18	93
Female	0.56	0.50	0	1
Non-white Race	0.33	0.47	0	1
Married	0.56	0.50	0	1
Children under 18	0.25	0.44	0	1
Family Income	6.06	3.36	1	16
Republican	0.34	0.47	0	1
Independent	0.19	0.40	0	1
Perceptions about Severity of Climate Change	2.71	1.09	1	4
Risk Aversion	5.24	2.42	0	10
Trust in Powerline Companies	3.19	0.94	1	5
Trust in Government Factor	0.0000000033	1.00	-1.93	2.84
Government Makes Quality Energy Decisions	2.96	0.80	1	5
New Environmental Paradigm Factor	-0.0000000027	1.00	-2.99	1.76
Perceptions of Local Environmental Quality	2.81	0.76	1	4
Perceptions of Local Economic Quality	2.55	0.79	1	4
General Support for Solar	4.11	1.10	1	5
General Support for Wind	3.99	1.17	1	5
General Support for Natural Gas	3.59	1.09	1	5
General Support for Coal	2.71	1.37	1	5
General Support for Transmission Lines	3.49	1.00	1	5

The results of the ordered probit models are presented in Table 6. Models 1 and 2 pertain to the renewable energy treatment, Models 3 and 4 to the natural gas treatment, and Models 5 and 6 to the coal treatment. The “treatment” variable is a binary variable that indicates whether the respondent was in the treatment group. For each treatment group, we first present a model that controls for alternative explanations offered by the extant literature, but does not include a measure of general support for both the generation source (e.g., solar, wind, natural gas, or coal) or transmission lines. In the second model for each, we add these relevant variables (e.g., wind, solar, and transmission line support for the renewable energy treatment).

**Table 6:** Ordered probit regression results (dependent variable = level of support for transmission line near one’s home).

	Model 1	Model 2	Model 3	Model 4	Model 5	Model 6
	RE Treatment		NG Treatment		Coal Treatment	
**Treatment**	0.332***	0.298***	-0.178**	-0.235***	-0.434***	-0.540***
	(0.0754)	(0.0763)	(0.0742)	(0.0754)	(0.0744)	(0.0759)
**Alternative Explanations for Support**						
Perceptions about Severity of Climate Change	0.0558	-0.0000211	-0.0431	-0.00684	-0.145***	0.000441
	(0.0501)	(0.0522)	(0.0496)	(0.0504)	(0.0484)	(0.0514)
Risk Aversion	0.0523***	0.0551***	0.0452***	0.0434***	0.0506***	0.0415***
	(0.0158)	(0.0160)	(0.0162)	(0.0164)	(0.0156)	(0.0159)
Trust in Powerline Companies	0.161***	0.0675	0.138***	-0.0119	0.176***	0.0487
	(0.0489)	(0.0509)	(0.0470)	(0.0496)	(0.0469)	(0.0491)
Trust in Government Factor	-0.00281	-0.000911	0.0714	0.0837*	0.123***	0.0857*
	(0.0478)	(0.0484)	(0.0447)	(0.0454)	(0.0473)	(0.0481)
Government Makes Quality Energy Decisions	0.159***	0.108*	0.135**	0.0859	-0.000157	-0.00915
	(0.0552)	(0.0563)	(0.0545)	(0.0555)	(0.0532)	(0.0540)
Monthly Expenditures on Electricity	0.205***	0.171**	0.192***	0.153**	0.0911	0.0990
	(0.0728)	(0.0709)	(0.0706)	(0.0711)	(0.0698)	(0.0694)
New Environmental Paradigm Factors	0.0968*	0.00661	-0.136***	-0.0935*	-0.161***	-0.0694
	(0.0510)	(0.0548	(0.0505	(0.0514	(0.0504	(0.0526
Perceptions of Local Environmental Quality	-0.0310	-0.0171	0.104*	0.0454	0.0499	0.0155
	(0.0628)	(0.0641)	(0.0612)	(0.0623)	(0.0615)	(0.0624)
Perceptions of Local Economic Quality	0.116**	0.132**	0.00482	0.0144	-0.0666	-0.0436
	(0.0559)	(0.0565)	(0.0564)	(0.0573)	(0.0572)	(0.0578)
General Support for Solar		0.166***				
		(0.0538)				
General Support for Wind		0.146***				
		(0.0516)				
General Support for Natural Gas				0.244***		
				(0.0429)		
General Support for Coal						0.302***
						(0.0397)
General Support for Transmission Lines		0.314***		0.326***		0.305***
		(0.0429)		(0.0464)		(0.0441)
**Demographics**						
Age	0.00226	0.000303	0.00511*	0.000680	0.0000102	-0.000378
	(0.00271)	(0.00274)	(0.00263)	(0.00271)	(0.00259)	(0.00262)
Female	-0.271***	-0.125	-0.284***	-0.107	-0.319***	-0.186**
	(0.0792)	(0.0817)	(0.0797)	(0.0826)	(0.0774)	(0.0805)
Non-white Race	-0.121	-0.131	-0.0374	-0.0229	0.0106	-0.0423
	(0.0889)	(0.0897)	(0.0894)	(0.0904)	(0.0874)	(0.0885)
Married	0.00678	0.00744	-0.0780	-0.0737	0.0663	0.0304
	(0.0841)	(0.0850)	(0.0828)	(0.0838)	(0.0837)	(0.0848)
Children under 18	-0.00794	-0.0366	-0.0238	-0.0845	-0.0656	-0.0681
	(0.0978)	(0.0987)	(0.0940)	(0.0951)	(0.0912)	(0.0922)
Family Income	0.000528	-0.00374	0.0171	0.00725	0.00734	0.00984
	(0.0122)	(0.0123)	(0.0127)	(0.0129)	(0.0126)	(0.0128)
Republican	0.0714	0.0223	0.198*	0.132	0.273***	0.107
	(0.106)	(0.107)	(0.105)	(0.106)	(0.106)	(0.108)
Independent	-0.190*	-0.188*	0.0298	-0.0347	0.135	0.0507
	(0.109)	(0.111)	(0.107)	(0.109)	(0.105)	(0.107)
Cutpoint 1	0.00709	1.673***	-0.502	0.428	-1.187***	0.361
	(0.353)	(0.399)	(0.351)	(0.366)	(0.343)	(0.376)
Cutpoint 2	0.468	2.172***	0.204	1.192***	-0.700**	0.891**
	(0.351)	(0.399)	(0.347)	(0.363)	(0.340)	(0.374)
Cutpoint 3	1.549***	3.325***	1.510***	2.580***	0.391	2.061***
	(0.353)	(0.404)	(0.348)	(0.367)	(0.339)	(0.377)
Cutpoint 4	2.456***	4.297***	2.580***	3.745***	1.334***	3.094***
	(0.357)	(0.411)	(0.354)	(0.376)	(0.343)	(0.384)
N	834	833	848	845	851	849

Standard errors in parentheses

* p<0.10

** p<0.05

*** p<0.01

We do not include survey weights in our statistical analysis. As argued by Miratrix et al. [61], when using a broadly representative and high-quality sample such as one obtained through YouGov, as we do here, including weights does not lead to sufficiently different statistical results but it does come at the expense of a loss of statistical power. As a robustness check, we also estimate these regression models with weights included, although not shown (available upon request), and the results do not differ substantially.

Results reveal that the treatment effect persists, even after including an extensive list of controls that reflect factors that have already been empirically shown to drive support for energy facilities. It is especially noteworthy that the treatment effect remains statistically significant even when controlling for one’s general support for transmission lines as well as renewables, natural gas, and coal, respectively. This result highlights that, when presented with the vignette, respondents did not simply base their support for a transmission line on their general level of support with transmission lines or specific energy types. Rather, the vignette elicited a response likely rooted in deeper emotional sentiment about the source of energy and its associated attributes, and based in intuitive reasoning. The fact that the vignette noted that the transmission lines would be located near one’s home also likely bolstered this affective reasoning response.

Not surprisingly, other factors are important as well, such as one’s risk aversion and general support for specific energy resources and transmission lines. Many variables are statistically significant before we control for general support of transmission lines, but then lose significance after we control for general levels support.

## Implications and discussion

Based on a survey experiment, our analysis reveals that, even though all transmission lines carry electrons (and carry all electrons according to loop flow dynamics), and an electron from one source of energy is no different than an electron from another source, individuals’ support for a new transmission line is shaped by their understanding of the source of the electrons. More specifically, if an individual is informed that the electricity is sourced from wind or solar, that individual will be significantly more supportive of building new local transmission lines than if they were not informed about the source of electricity. In contrast, if an individual is informed that the electricity is sourced from natural gas or coal, she or he will be significantly less likely to support a new build than if told nothing about the source. This effect is especially large and negative in magnitude in the case of coal production.

It is perhaps seemingly irrational to have varying levels of support for the same hypothetical transmission line, depending on the source of energy to which the line connects. The social psychology literature, however, lends insights about why the individuals in our sample may have responded to the vignette experiments in the way that they did. Specifically, we pull from literature on cognitive heuristics and the law of contagion as a possible explanation for respondents’ reactions to the vignettes. According to the law of contagion, when one forms a strong emotional sentiment toward one thing, and that thing comes into contact or association with another thing, the emotion is transferred to it as well. In the present case, emotions such as possible disgust with coal—or even the attributes that, in one’s mind, comprise coal—transfer to the electricity line. Since we told respondents that the line would be located near them, this affective response was likely even stronger than had we simply asked about transmission lines in general.

The contribution of this analysis is threefold. First, the research design—which relies on a randomized experiment and facilitates comparison across energy types—is an improvement upon previous studies that tend to focus exclusively on single cases and secondary data. Second, this study demonstrates, or at least suggests the possibility, that people use cognitive heuristics to determine their comfortability with and their support for energy infrastructure. Third, this study has significant practical implications, given the importance of public support for energy infrastructure development, and the broader context of the energy transition from fossil fuels toward renewable energy that the world is currently experiencing.

These results also have important practical implications for the siting of new transmission lines and new power plants. Greater opposition to transmission infrastructure can result in significant delays of project approval, at best, and termination of the project, at worst. Given plans in the U.S. to inject substantial investments in new renewable energy and natural gas plants, which will require the expansion of current transmission infrastructure, our results suggest that citizen support will be mixed. If citizens are aware—or at least think—that new transmission lines are installed specially to connect new wind and solar developments, their support will be much higher than if they are not aware. These implications may also hold for other types of energy infrastructure that will be an integral part of the energy transition, such as smart-meters, if consumers connect them mentally to certain sources of energy.

Our results also suggest that transmission line developers may garner greater support from communities that will host such lines if they explain explicitly that at least one source of electricity is solar or wind. Of course, higher support for transmission lines that carry renewable electricity may not be sufficient to overcome all opposition, especially from property owners most directly affected by the siting; nor is it guaranteed that a response to a hypothetical survey scenario reflects how one would respond to the same scenario in reality. Moreover, the weight that people give to the environmental attributes of the power generation source may vary across the geographic footprint of a power line. This may be especially true in cases of long-range transmission, where much of the line will be located far away (i.e., hundreds of miles) from the generating source. These limitations notwithstanding, the results of this study highlight the importance of messaging during transmission line planning and development.

There are few coal plants in the U.S. that are planned for construction over the next ten years. Yet, in the event that the repeal of the Clean Power Plan, the rejection of the Paris Agreement, or a possible introduction of new coal supporting policies lead developers to consider new coal power plants again, our results suggest that transmission lines built to connect coal generation to load centers may be marked by significant opposition, on top of the opposition that coal power plants are likely to face as well [15].
